# Supplementary material for: Quantum dynamics simulation of exciton-polariton transport
Source: Nat Commun. 2025 Jul 1;16:5431. doi: 10.1038/s41467-025-61298-9 (PMC12218039; doi:10.1038/s41467-025-61298-9)
Supplement: Supplementary file 1 — Supplementary information [file 41467_2025_61298_MOESM1_ESM.pdf]

Supplementary Information for: Quantum dynamics simulation of exciton-polariton transport

Niclas Krupp,[*,†] Gerrit Groenhof,[‡] and Oriol Vendrell[*,†]

†*Theoretische Chemie, Physikalisch-Chemisches Institut, Universität Heidelberg, INF 229, 69120 Heidelberg, Germany*

‡*Nanoscience Center and Department of Chemistry, University of Jyväskylä, P.O. Box 35, Jyväskylä 40014, Finland*

E-mail: niclas.krupp@pci.uni-heidelberg.de; oriol.vendrell@uni-heidelberg.de

Supplementary Note 1. Convergence of Transport Simulations

The spatial separation Δx between individual molecules and the Rabi splitting $\hbar\Omega_R$ meV are treated as fixed parameters of our quantum dynamical simulations. This leaves the numbers of cavity modes (M) and molecules (N) as convergence parameters of our model. Note that cavity coupling strengths $g_j(k_x)$ need to be scaled by $1/\sqrt{N}$ to keep the Rabi splitting constant. We choose $\hbar\Omega_R = 328\,\text{meV}$ and $\Delta x = 250\,\text{nm}$. The number of cavity modes M determines the maximum cavity-mode wavevector $k_{x,\text{max}} = 2\pi M/L$ (and equivalently the maximum cavity-mode energy), whereas the number of molecules N determines the quantization length $L = N\Delta x$ and thus the resolution of cavity modes in k-space via $\Delta k_x = 2\pi/L$.[1]

In Supplementary Fig. 1 we investigate the effect of varying N and M for a model without vibronic coupling ($\kappa = 0\,\text{meV}$) and without static disorder ($\sigma = 0\,\text{meV}$). Our findings are very

similar to the convergence study in Ref.[2]. Supplementary Fig. 1a,c show that a sufficiently large number of molecular sites is required to capture the spatial expansion of the propagating polariton. Otherwise, an artificial localization is found (cf. $N = M = 64$ and $N = M = 128$ in Supplementary Fig. 1a). Moreover, a broad range of cavity modes contributes to the transport dynamics, making the inclusion of higher-energy modes mandatory to obtain converged results (cf. Supplementary Fig. 1b,d).

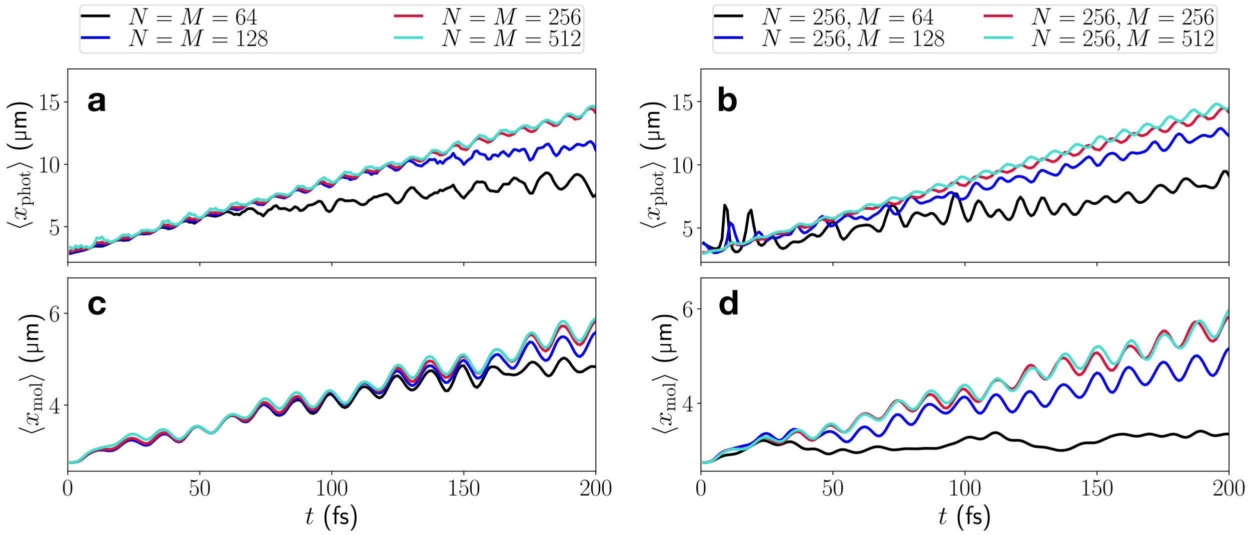

Supplementary Figure 1: Convergence study. Mean positions of photonic (a,c) and molecular subsystems (b,d) for various numbers of cavity modes (M) and molecules (N) while keeping the intermolecular distance Δx fixed. The propagation is initiated by sudden excitation of a single molecule located at $x = 2.5\,\mu\text{m}$.

Next, we examine the impact of increasing the molecular density, i.e. the number of molecules per quantization length, N/L. To this end, we keep the previously determined quantization length $L = 256 \cdot 250\,\text{nm}$ as well as the Rabi splitting fixed, and double the number of molecules by choosing $\Delta x = 125\,\text{nm}$. Doubling the molecular density has no substantial effect on the propagation of the photonic subsystem, for both $M = 256$ and $M = 512$. The overall propagation behavior of the molecular subsystem changes little as well, consisting of a stationary population at the initially excited position and a wavefront propagating at identical $v_{\text{gr}}^{\text{LP,max}} = 67.9\ \mu\text{m}\,\text{ps}^{-1}$ in all cases. Note that the intensity of the stationary feature increases when doubling the molecular density, while the propagating

wavefront is unaffected. This is due to the increased number of non-propagating highly-molecular states which are initially populated by off-resonant excitation.

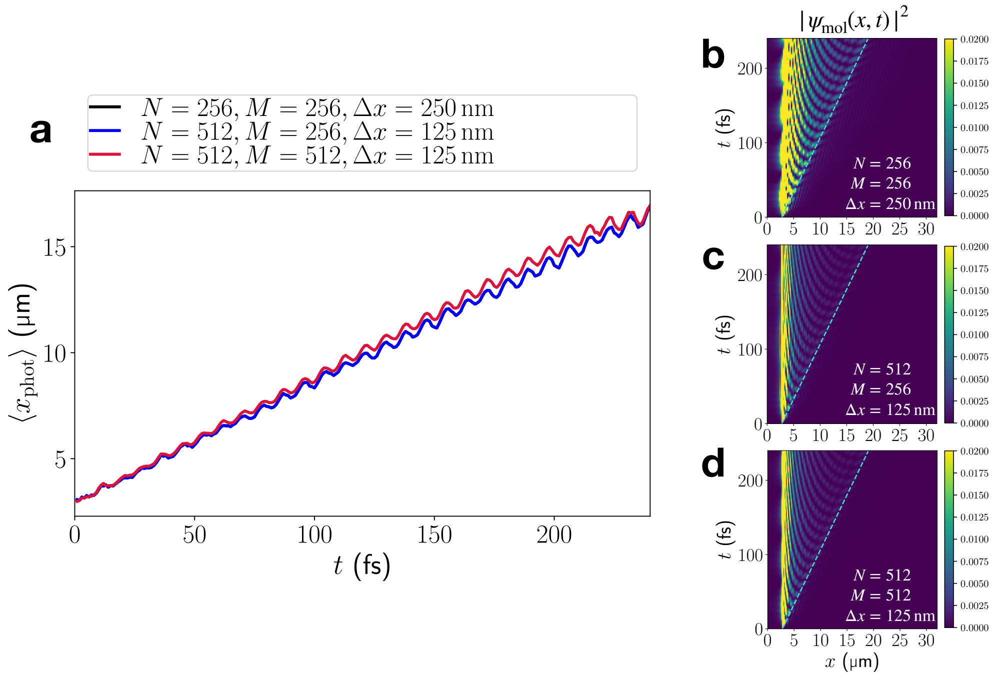

Supplementary Figure 2: Impact of higher molecular density. (a) Mean photonic position $\langle x_{\mathrm{phot}} \rangle (t)$ and (b)-(d) molecular real-space density $|\psi_{\mathrm{mol}}(x, t)|^2$ for $\kappa = 0\,\mathrm{meV}$. N, M and intermolecular distances Δx are varied. The propagation is initiated by sudden excitation of a single molecule located at $x = 2.5\,\mathrm{\mu m}$. Cyan dashed lines in (b)-(d) indicate linear motion at the maximum group velocities of LPB ($67.9\,\mathrm{\mu m\,ps^{-1}}$).

Since the rotating wave approximation (RWA) is not used in the present simulations, states beyond the single-excitation subspace (SES) may participate. Other theoretical studies on polariton transport have employed the RWA (e.g. Ref.[3]). The summed population of the ground-state and SES is inspected in Supplementary Fig. 3 to assess the validity of the RWA in the present model. Various laser pulses which resonantly excite to the LPB or UPB are considered. Minimal leakage ($\leq 1\%$) to higher-excitation spaces is found in Supplementary Fig. 3b. Furthermore, the absence of Rabi-type oscillations in Supplementary Fig. 3a indicates that laser intensities are small enough to excite within the perturbative regime.

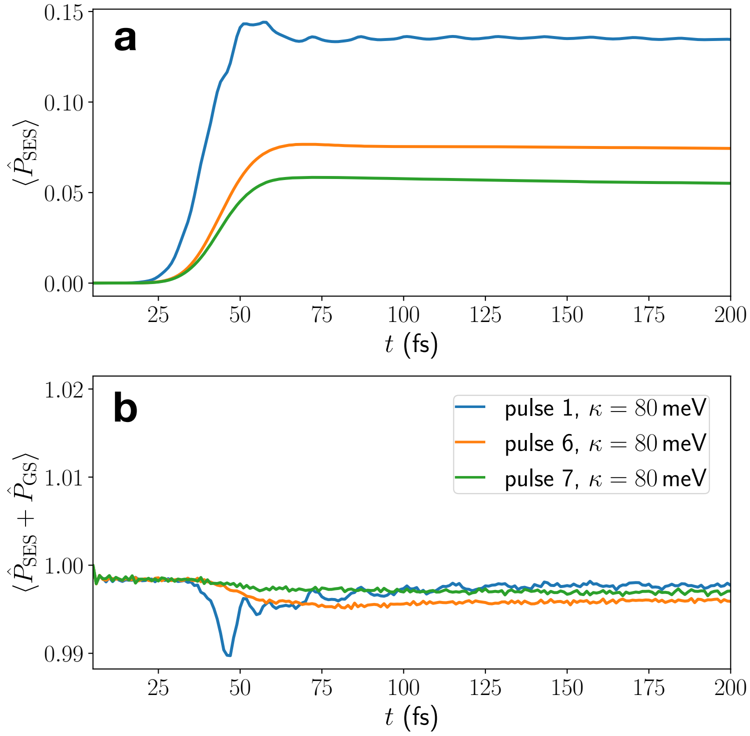

Supplementary Figure 3: (a) Single-excitation subspace (SES) population and (b) ground-state + SES population after laser excitation for three representative pulses. The SES population stays almost constant after the pulse is over, indicating that the dynamics predominantly occurs within the SES. Leakage to higher-excitation subspaces either due to a breakdown of the rotating wave approximation or high laser intensities is very small, the majority of population resides in the ground-state or SES.

Supplementary Note 2. Lossy cavity

Supplementary Fig. 4 shows the real-space propagation of a polariton wavepacket excited by a broad-band laser pulse which is targeting the UP at resonant wavevector k_x^{res}. Two cavity lifetimes $\tau_C = 12\,\text{fs}$ and $24\,\text{fs}$ are considered. This situation is akin to the experiment in Ref.[4]. Comparing simulations without (Supplementary Fig. 4a,b) and with (Supplementary Fig. 4c,d) vibronic coupling substantiates intramolecular vibronic interactions as a source for polariton wavepacket contraction. Vibronically-driven population transfer to stationary dark states together with the τ_C-dependent coherent propagation of UPB states leads to a cavity loss-dependent propagation distance, as discussed in the main text.

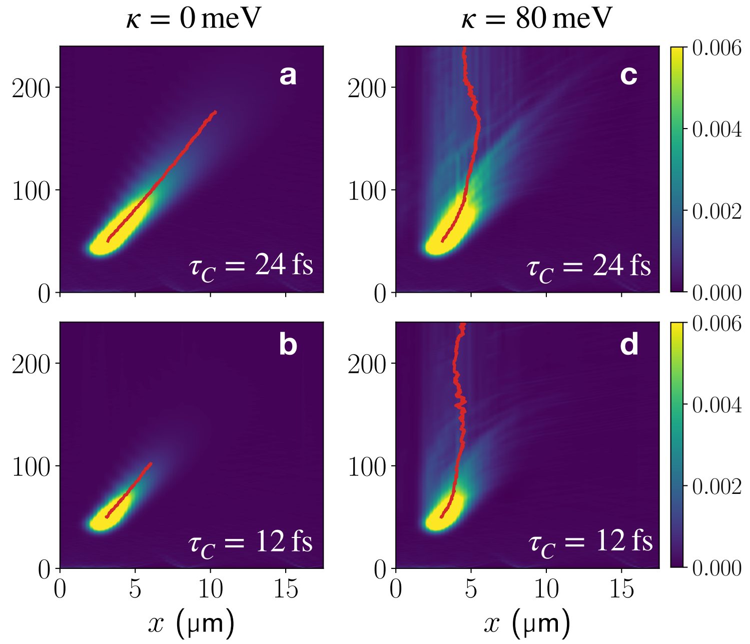

Supplementary Figure 4: Polariton wavepacket contraction in lossy cavities. Excitation with broad-band laser pulse ($F_t = 10\,\text{fs}$, $k_x^{(0)} = k_x^{\text{res}}$, $\omega_L = 4.351\,\text{eV}$) targeting UP at resonant wavevector. Without (a,b) and with (c,d) vibronic coupling for two cavity mode lifetimes τ_C.

Supplementary Note 3. Disorder realizations

To obtain the results with static disorder (cf. Fig. 5 in main text), molecular electronic excitation energies ω_m are sampled from a Gaussian distribution with $\sigma_m = 30\,\text{meV}$ around

the mean $\bar{\omega}_m = 4.18\,\text{eV}$. Each MSD and density presented in Fig. 5 of the main text is the average over 5 such realizations. The results of the individual propagations are shown in Supplementary Fig. 5.

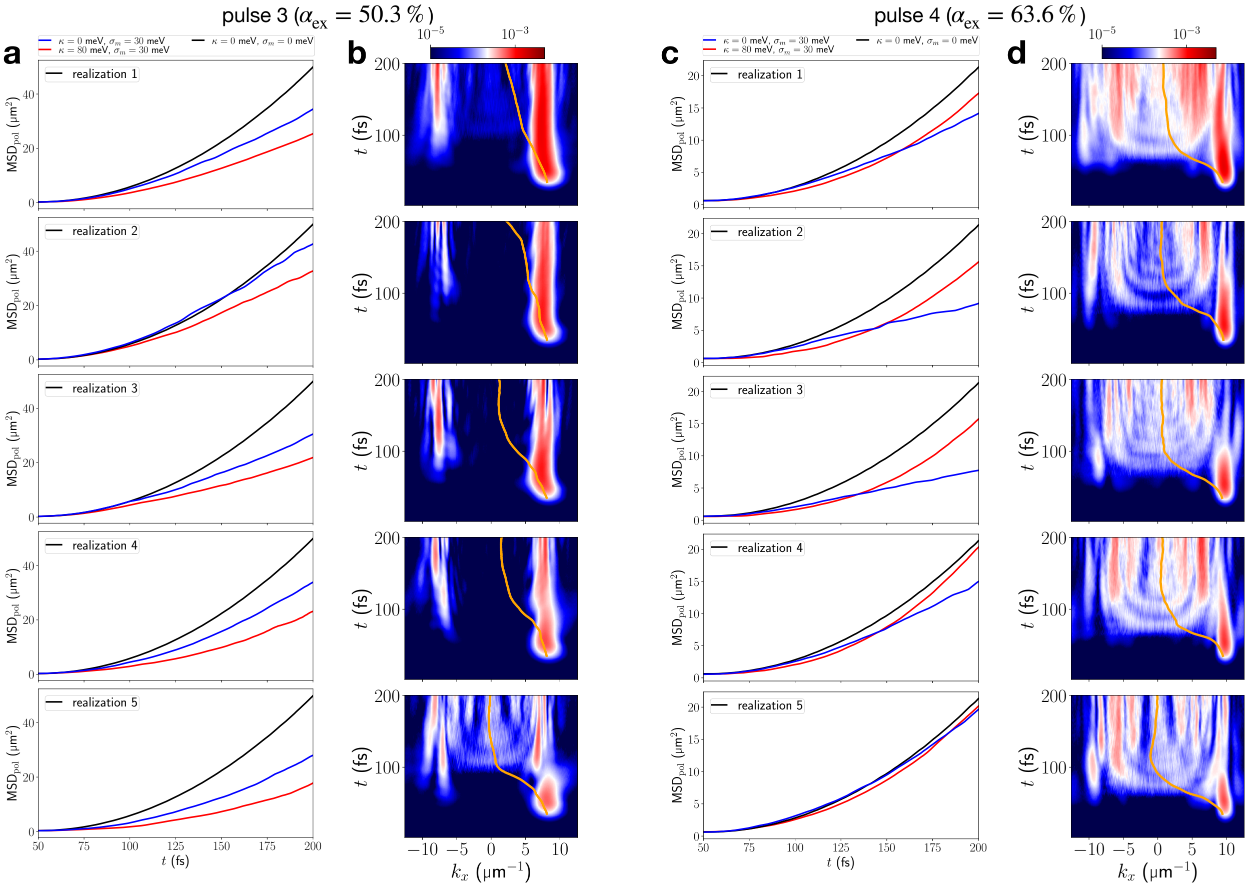

Supplementary Figure 5: Individual realization of disordered molecular ensembles, gaussian distribution of ω_m's with $\sigma_m = 30\,\text{meV}$.

Supplementary References

(1) Ribeiro, R. F. *Commun. Chem.* **2022**, *5*, 48.

(2) Aroeira, G. J.; Kairys, K. T.; Ribeiro, R. F. *J. Phys. Chem. Lett.* **2023**, *14*, 5681–5691.

(3) Sokolovskii, I.; Tichauer, R. H.; Morozov, D.; Feist, J.; Groenhof, G. *Nat. Commun.* **2023**, *14*, 6613.

(4) Pandya, R.; Ashoka, A.; Georgiou, K.; Sung, J.; Jayaprakash, R.; Renken, S.; Gai, L.; Shen, Z.; Rao, A.; Musser, A. J. *Adv. Sci.* **2022**, *9*, 2105569.
